# Alpha-Latrotoxin Rescues SNAP-25 from BoNT/A-Mediated Proteolysis in Embryonic Stem Cell-Derived Neurons

**DOI:** 10.3390/toxins3050489

**Published:** 2011-05-13

**Authors:** Mariano Mesngon, Patrick McNutt

**Affiliations:** United States Army Medical Research Institute of Chemical Defense, 3100 Ricketts Point Road, Gunpowder, MD 21010, USA; Email: patrick.mcnutt@us.army.mil

**Keywords:** botulinum neurotoxin, alpha-latrotoxin, embryonic stem cell-derived neurons

## Abstract

The botulinum neurotoxins (BoNTs) exhibit zinc-dependent proteolytic activity against members of the core synaptic membrane fusion complex, preventing neurotransmitter release and resulting in neuromuscular paralysis. No pharmacologic therapies have been identified that clinically relieve botulinum poisoning. The black widow spider venom α-latrotoxin (LTX) has the potential to attenuate the severity or duration of BoNT-induced paralysis in neurons via the induction of synaptic degeneration and remodeling. The potential for LTX to antagonize botulinum poisoning was evaluated in embryonic stem cell-derived neurons (ESNs), using a novel screening assay designed around the kinetics of BoNT/A activation. Exposure of ESNs to 400 pM LTX for 6.5 or 13 min resulted in the nearly complete restoration of uncleaved SNAP-25 within 48 h, whereas treatment with 60 mM K^+^ had no effect. Time-lapse imaging demonstrated that LTX treatment caused a profound increase in Ca^2+^ influx and evidence of excitotoxicity, though ESNs remained viable 48 h after LTX treatment. This is the first instance of a cell-based treatment that has shown the ability to eliminate BoNT activity. These data suggest that LTX treatment may provide the basis for a new class of therapeutic approach to BoNT intoxication and may contribute to an improved understanding of long-term mechanisms of BoNT intoxication and recovery. They further demonstrate that ESNs are a novel, responsive and biologically relevant model for LTX research and BoNT therapeutic drug discovery.

## 1. Introduction

The *Clostridium botulinum* neurotoxins (BoNTs) are the most poisonous substances known, with human toxicities estimated to be as low as 1–2 ng/kg [1]. Following ingestion, inhalation or injection, BoNTs gain access to the presynaptic termini of neuromuscular junctions and specifically target the soluble *N*-ethylmaleimide-sensitive factor attachment protein receptor (SNARE) proteins VAMP-2, SNAP-25, or syntaxin-1 for proteolysis [2]. In the standard model of synaptic neurotransmission, the arrival of an action potential (AP) at the presynaptic compartment triggers Ca^2+^ influx through voltage-gated channels, which in turn induces the synaptic SNARE complex to mediate synaptic vesicle fusion and neurotransmitter release. Consequently, BoNT cleavage of these proteins leads to inhibition of synaptic exocytosis and resulting in skeletal muscle paralysis with emergent respiratory failure [1]. Although passive immunotherapy can reduce vascular toxin load, once the toxin is sequestered within the presynaptic terminus there are currently no therapeutic approaches that restore normal synaptic activity. Interference with toxin internalization or activation involves a short therapeutic window; delays the onset of paralysis but does not prevent intoxication; and does not appear to add significant clinical value to the current post-exposure prophylaxis offered by passive immunizations [3,4]. Thus, once evidence of intoxication is present, clinical options are largely limited to supportive care [5]. Depending on the BoNT serotype, paralysis can persist for months, requiring sustained intensive medical care [6]. Furthermore, once the toxin is cleared from poisoned nerve termini, the synapse must be regenerated and coordinated neuromuscular control re-established [7,8,9]. For these reasons, the BoNTs present potent health risks, and in recognition of their disruptive potential the neurotoxins have been designated as one of six CDC Category A bioterrorism agents. 

The neurotoxin α-latrotoxin (LTX) is a highly potent secretagogue derived from the venom of the black widow spider (*Latrodectus mactans tredecimguttatus)* that induces fulminant neurotransmitter release at central and autonomic synapses [10,11,12]. In the most direct method of action, LTX inserts into the membrane following binding to the cell surface proteins neurexin or latrophilin and forms homotetramers with a central, non-selective cation-conducting pore [10,13,14,15]. The resultant influx of Ca^2+^ into the synaptic terminal induces sustained synaptic exocytosis, mimicking the activation of voltage-dependent Ca^2+^ channels during an AP. Surprisingly, this mechanism supports high levels of neurotransmitter release even in the absence of SNAP-25, synaptobrevin-2 or Munc13-1, which under normal circumstances nearly eliminates Ca^2+^-evoked vesicle fusion [16,17,18,19]. LTX treatment caused a rapid, prolonged release of large amounts of neurotransmitter, followed by dose-dependent changes in nerve terminal morphology, presumably as a consequence of excitotoxicity [20,21,22,23]. *In vivo*, treatment of neuromuscular junctions with a crude homogenate from black widow spider glands results in structural and functional degeneration in hours, followed by regeneration of neuromuscular junctions and resumption of synaptic transmission at the original endplate within days [23,24]. Treatment of BoNT/A-intoxicated extensor digitalis longus (EDL) neuromuscular junctions with the same crude preparation shortened paralysis from weeks to days [25]. Similarly, recovery from BoNT intoxication can also be expedited by nerve crush treatment [26,27]. Together, these findings suggest that the use of chemical or physiological treatments that induce synaptic regeneration of remodeling may accelerate the recovery of nerve terminals from BoNT paralysis. 

We have previously described a method to generate effectively pure cultures of glutamatergic neurons (ESNs) from suspension-cultured embryonic stem (ES) cells within 8 days, with typical yields of approximately 7 × 10^8^ glutamatergic neurons from 4 × 10^6^ mES cells [28]. ESNs express and correctly localize neuron-specific proteins, form synapses and release glutamate in a calcium-dependent manner under depolarizing conditions. The BoNT substrate SNARE proteins are expressed within 5 d of plating, and treatment with 0.81 pM BoNT/A holotoxin results in proteolysis of 50% of cellular SNAP-25 within 24 h. This sensitivity to BoNT/A is within two-fold of that observed in primary spinal cord neurons after 48 h of exposure, suggesting that botulinum is internalized, processed and behaves similarly in the two cell models [29]. In this effort, we developed ESNs as a therapeutic research platform, hypothesizing that LTX activity in the presynaptic compartment has potential as a research tool and a novel therapeutic approach for botulinum intoxication. We report that ESNs are responsive to the acute and long-term consequences of LTX treatment; *in vivo* reports of accelerated nerve terminal regeneration following administration of crude gland homogenates could be attributed specifically to LTX activity; and LTX treatment results in the recovery of full-length SNAP-25 within 48 h.

## 2. Materials and Methods

### 2.1. Reagents

Botulinum holotoxin type A (BoNT/A) (Metabiologics, Madison, WI, USA) was resuspended in phosphate buffered saline, pH 7.4 to 1 mg/mL, and stored at −20 °C. Latrotoxin (Sigma-Aldrich, St. Louis, MO, USA) was resuspended to 300 nM in H_2_O and stored at −20 °C. Fluo-4 (Invitrogen, Carlsbad, CA, USA) and Calcein/AM (Invitrogen) were prepared per the manufacturer’s instructions. During time-lapse imaging neurons were maintained in basal electrophysiologic buffer (BEB; 10 mM glucose, 1 mM MgCl_2_, 10 mM HEPES, 2 mM CaCl_2_, 3 mM KCl, 136 NaCl and 0.1% BSA, pH 7.4, 310 ± 10 mOsm). High potassium electrophysiologic buffer (KEB) was prepared similarly, except with 60 mM KCl and 79 mM NaCl.

### 2.2. Embryonic Stem Cell Culture and Neuronal Differentiation

Murine embryonic stem cells were maintained and differentiated into ESNs as described [28]. ESNs were plated in PDL-coated 60 mm dishes at 125,000 cells/cm^2^ or PDL- and laminin-coated 18 mm coverslips at 100,000 cells/cm^2^ and maintained in Neurobasal-A medium (NBA) with B27 vitamins (Invitrogen, Carlsbad, CA, USA).

### 2.3. Immunoblotting

ESN cultures were washed with 2 mL PBS, lysed by addition of 250 µL of denaturing cell lysis buffer (Sigma-Aldrich) and harvested by scraping. Lysates were vortexed briefly, stored at 4 °C for 30 min and clarified by centrifugation for 5 min through a Qiashredder (Qiagen, Valencia, CA, USA) at 16,000 xg. Total protein concentration was determined by bicinchoninic acid (BCA) analysis (Thermo Scientific, Rockford, IL, USA), and 15 μg of total protein was separated on a 12% Nupage gel (Invitrogen) with MOPS running buffer. Gels were transferred to PVDF and probed with a mouse anti-SNAP-25 antibody (Abcam, Cambridge, MA, USA) and a mouse anti-syntaxin-1a antibody (Abcam), both diluted 1:1000 in TBS Superblock with 0.05% Tween-20 (TBST, Invitrogen). Bands were visualized with goat anti-mouse Alexa-488 diluted 1:2500 in TBST and imaged with a Versadoc MP4000 (Biorad, Hercules, CA, USA).

### 2.4. Time-Lapse Confocal Microscopy

Images were collected on a Zeiss LSM-700 confocal microscope with constant-temperature environmental chamber. For Fluo-4 staining, ESNs on 18-mm coverslips were loaded with 1 μM Fluo-4 for 20 min and washed thoroughly. Coverslips were mounted in a Warner (Hamden, CT) closed-bath imaging chamber, maintained at 37 °C with a heated stage and perfused with phenol-free Hibernate (Brainbits, Springfield, IL). For calcein green staining, cells were incubated with 1 µM calcein green in NBA for 30 min, then washed thoroughly and mounted as above. In both cases, coverslips were imaged at 63× using the 488 laser and manufacturer recommended filter sets. 

## 3. Results and Discussion

### 3.1. Results

#### 3.1.1. Optimization of the Screening Model

**Figure 1:**
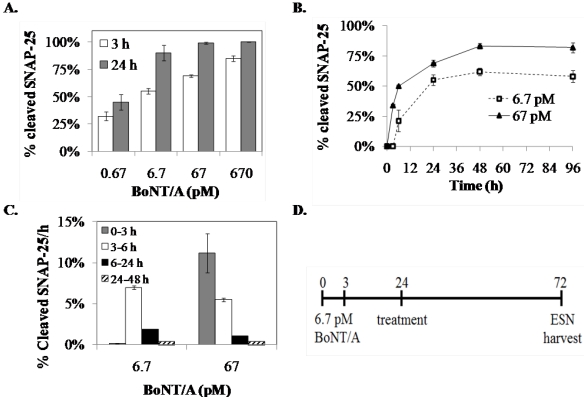
Kinetics of SNAP-25 cleavage following internalization of BoNT/A under different conditions. (**A**) DIV 25-31 ESNs in 6 cm dishes were treated with 0.67–670 pM BoNT/A in B27-NBA medium for 3 h (white columns) or 24 h (gray columns). For the 3 h treatment, cells were washed twice to remove toxin at 3 h and incubated for an additional 21 h. All treatments were harvested at 24 h and the percent of cleaved SNAP25 was determined by densitometry of western blots; (**B**) Summary of SNAP-25 cleavage in embryonic stem cell-derived neurons (ESNs) between 24 to 96 h after a 3 h exposure to 6.7 or 67 pM BoNT/A; (**C**) Evaluation of the rate of SNAP-25 cleavage, measured as percent-cleaved SNAP-25 per hour, averaged across each time point. For all experiments, *n* = 5 or more replicates; (**D**) Drug discovery treatment paradigm designed around kinetics of BoNT/A internalization and SNAP-25 cleavage in ESNs.

Previously we reported that 0.81 pM BoNT/A treatment for 24 h results in cleavage of 50% of SNAP-25 in ESNs [28]. Reasoning that application of higher doses for a shorter period might accelerate toxin internalization, we exposed ESNs to 0.67–670 pM BoNT/A for 3 h or 6 h and evaluated SNAP-25 cleavage after 24 h (Figure 1A). In comparing the percent cleaved SNAP-25 at 24 h following either 3 or 24 h intoxication, we found that roughly three-quarters of toxin internalization occurs within the first few hours. Since both 6.7 and 67 pM produced roughly 50% cleaved SNAP-25 after 3 h exposure, we longitudinally evaluated BoNT/A activity from 3–96 h (Figure 1B). At each concentration the rate of SNAP-25 cleavage per hour slowed dramatically after 24 h, suggesting a balance between rates of SNAP-25 synthesis and cleavage (Figure 1C). Based on these data, we designed a screening assay in which ESNs were exposed to 6.7 pM BoNT/A for 3 h, then washed and incubated for 21 h to allow full toxin activation. Candidate therapeutics were applied at 24 h, and the recovery of full-length SNAP-25 was evaluated 48 h after therapeutic treatment (summarized in Figure 1D). The duration of incubation following treatment was selected based on reports that approximately 2% of cellular SNAP-25 is recycled per hour [30].

#### 3.1.2. LTX Treatment of BoNT/A-Treated ESNs Restores Full-Length SNAP-25 Protein within 48 h.

**Figure 2:**
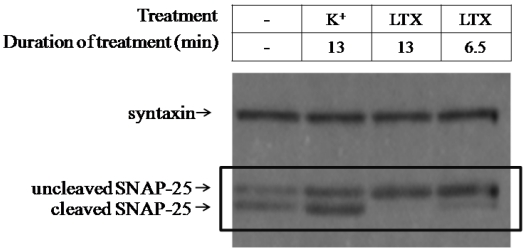
LTX but not K^+^ rescues SNAP-25 expression within 48 h in BoNT/A-intoxicated ESNs. BoNT/A-intoxicated ESNs were treated with 60 mM K^+^ or 400 pM LTX for designated times, and SNAP-25 cleavage was evaluated after 48 h by densitometry of western blots. Syntaxin is shown as a loading control.

To evaluate whether LTX treatment altered light chain (LC)/A activity in ESNs, BoNT/A-intoxicated ESNs were exposed to 400 pM LTX for 6.5 or 13 min, and SNAP-25 integrity was evaluated after 48 h. As a control, ESNs were also treated with 60 mM K^+^ (KEB) for 1.5 min, which evokes Ca^2+^-dependent glutamate release [28]. LTX treatment resulted in rescue of 92 ± 4.6% and 98 ± 1.7% (6.5 and 13 min, respectively) of full-length SNAP-25 within 48 h, whereas KEB treatment showed no difference from untreated neurons (Figure 2). The restoration of uncleaved SNAP-25 indicates that some aspect of LTX treatment results in the inactivation or clearance of LC/A from synaptic termini. Furthermore, these data suggest that latrotoxin may be the active moiety in experiments demonstrating that the administration of crude homogenate from black widow spider venom glands to BoNT/A-paralyzed neuromuscular junctions dramatically accelerates recovery [25].

#### 3.1.3. LTX Treatment Results in Prolonged Ca^2+^ Internalization

**Figure 3:**
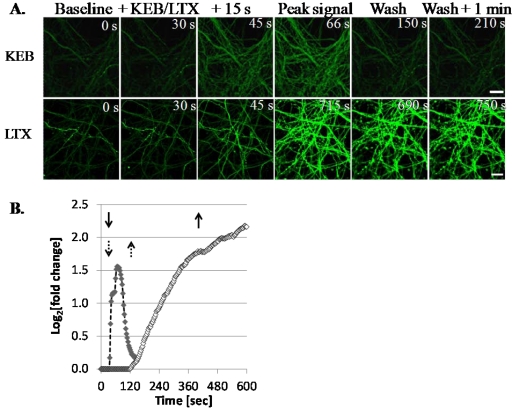
Kinetics of acute Ca^2+^ influx mediated by treatment with LTX or K^+^. (**A**) Increased Ca^2+^ influx (Fluo-4; green) peaked rapidly in response to 60 mM K**^+^** and returned to basal levels within 120 s (upper panels); 6.5 min treatment with 400 pM of LTX resulted in rapid increase of Ca^2+ ^with sustained intensity for over 570 s (lower panels); (**B**) Quantitative summary of Ca^2+^ response following addition (down arrows) of 60 mM K^+^ (closed diamonds; dashed arrows) or 400 pM LTX (open diamonds; solid arrows) from a single field of view. Whereas the K^+^-induced Ca^2+^ response returned to basal levels prior to washout (up arrows), the LTX-induced Ca^2+^ response continued to increase through 570 s. Scale bar = 10 µm.

LTX treatment incurs fulminant neurotransmitter release from primary neurons, partly in response to profound levels of Ca^2+^ internalization [31]. Since LTX treatment resulted in recovery of full-length SNAP-25 in ESNs, whereas KEB did not, we used the fluorescent intracellular calcium sensor Fluo-4 to compare the amplitude and duration of Ca^2+^ internalization evoked by these two treatments. Time-lapse confocal microscopy analysis of DIV 21 ESNs treated with KEB demonstrated an immediate rise in Fluo-4 fluorescence (Figure 3A). Fluorescence intensity increased within 15 s of KEB treatment, remained high for about 90 s and subsided prior to washout at 2.5 min (Figure 3B). Conversely, while ESNs treated with 400 pM LTX also showed a strong rise in Fluo-4 fluorescence, there were several key differences in the kinetics of the Ca^2+^ response. First, there was a 1.5 min delay between LTX addition and the onset of Fluo-4 fluorescence, suggesting that spider toxin requires time to bind synaptic receptors and form pores within the membrane at this concentration (Figure 3B). Unlike the self-limiting response from KEB treatment, LTX induced a steady increase in Fluo-4 signal throughout the experiment, even following rinses to remove residual toxin, demonstrating that LTX results in loss of cellular ionic homeostasis and that the KEB response was not limited by reduced levels of extracellular Ca^2+^. No apparent differences in onset, amplitude or duration were noted within the 20 min experimental window between the 6.5- and 13 min LTX treatment, nor did a 24 h intoxication with 67 pM BoNT/A prior to LTX or KEB treatment affect Ca^2+^ internalization (not shown).

#### 3.1.4. LTX-Treated ESNs Exhibit Evidence of Excitotoxicity That Partially Resolves between 24–48 h

**Figure 4:**
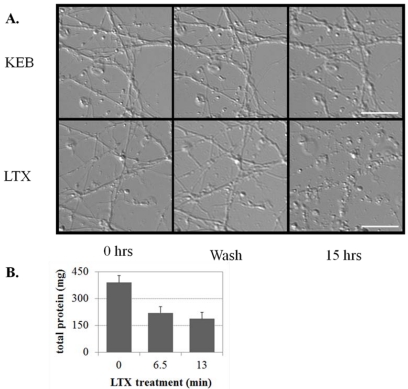
Comparison of neuronal morphology changes and total protein yield after treatment with K^+^ or LTX. (**A**) DIV 22 ESNs were treated with either 60 mM K^+^ (top) or 400 pM LTX in BEB (bottom), then perfused for 30 s with BEB. Differential interference contrast images were taken of the same field at the indicated time points. Scale bar = 10 µm; (**B**) Total protein yield (see methods) from cultures (*n* = 3) treated with 400 pM LTX for 6.5 or 13 min was decreased by approximately 50% relative to untreated cultures.

**Figure 5:**
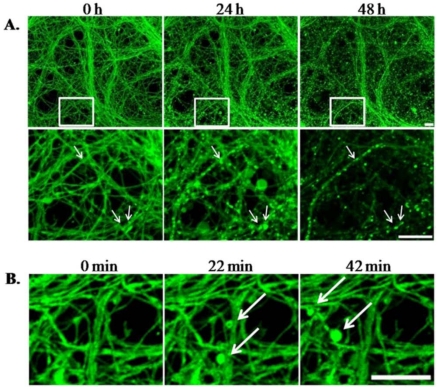
Examination of varicosity formation in calcein-labeled ESNs over time. (**A**). Top panels, low magnification image; bottom panels, high magnification of boxed area in upper panel. Arrows identify representative varicosities that appeared within 24 h and showed a substantive decrease in area by 48 h after treatment; (**B**) Varicosities appear as soon as 22 min after addition of LTX. All images were of the same field of view for all time points. Scale bar for all images = 10 µm.

Differential interference contrast images captured 15 h after treatment indicate that cultures treated with LTX for 6.5 min have large numbers of distributed varicosities and disrupted processes, whereas cultures treated with KEB for 13 min do not (Figure 4A). LTX treatment of ESNs decreased the total protein yield following cell lysis by 44 ± 16% and 52 ± 19% (6.5 and 13 min, respectively) compared to controls at 48 h after treatment (*p* < 0.01, *n* = 6; Figure 4B). We used calcein green staining to compare morphological changes in ESNs during the emergent and long-term response to LTX *versus *KEB. These observations are in agreement with results from LTX-treated spinal cord motoneurons and cerebellar granule neurons [31]. The development of axodendritic varicosities from existing processes was apparent within 30 min of LTX addition (Figure 5). Although neurons remained viable 24 h and 48 h after treatment, there was an overall decrease in viability and persistent evidence of varicosities. By 48 h some of these varicosities were resolved, suggesting that neuronal regeneration was underway.

### 3.2. Discussion

#### 3.2.1. Considerations in Developing a Screening Methodology

Although we previously demonstrated that neurons derived from embryonic stem cells have the potential of acting as a biologically relevant platform for botulinum research, we did not develop screening protocols to maximize the likelihood of rapidly identifying therapeutically useful candidates [28]. In this work we started by developing a cell-based assay using the kinetics of BoNT/A internalization and activation in ESNs to standardize the evaluation of therapeutic candidates.

Although it is a widespread practice to use toxin concentrations that are several orders of magnitude above the EC50 followed immediately by therapeutic administrations to expedite therapeutic screening assays, for several reasons this approach significantly increases the risk of failing to identify a valid therapeutic [32,33,34,35]. First, internalization of BoNT at high concentrations may result in broad cellular distribution as opposed to synaptic localization, altering the ability to evaluate therapeutic efficacy. Second, only a fraction of internalized toxin is capable of cleaving the majority of SNAP-25 within 6 h (see Figure 1). Thus, evaluating therapeutic candidates before all the internalized toxin is activated will artificially increase the ratio between therapeutic molecules and toxin, thereby increasing the apparent therapeutic activity. Finally, a therapeutic candidate applied too early may disrupt toxin processing rather than interfere with fully functional toxin. This latter point is particularly relevant in high-throughput screening approaches for post-exposure therapeutics in which libraries are selected without a mechanistic bias. 

In consideration of these concerns, we designed an assay around three principles intended to mitigate risks and ensure a high level of sensitivity for therapeutic efficacy. First, an incubation step was included between the removal of holotoxin and prior to therapeutic administration to allow internalized light chain to become fully activated. This step was designed to be sufficiently long to establish a steady state between cleavage of existing SNAP-25 and synthesis of new SNAP-25. Second, a dose was selected that would result in approximately 50% cleavage of SNAP-25 within the given time frame, yet be as low as possible to encourage toxin internalization and trafficking by synaptic endocytosis rather than non-specific endocytosis. Third, treatment efficacy timescales were selected around presumptive mechanisms of action. Thus, using dose response curves at multiple time points and exposure durations, we identified a 3 h exposure to 6.7 pM, with therapeutic application at 24 h and SNAP-25 evaluation at 72 h as the preferred model.

#### 3.2.2. LTX Rescue of Full-Length SNAP-25 Expression

The finding that LTX treatment of BoNT/A-intoxicated ESNs rescued full-length SNAP-25 expression is the first instance of a successful therapeutic application in derived neurons. This finding resulted from the inactivation of LC/A by an unknown mechanism, allowing newly synthesized SNAP-25 to accumulate without being proteolytically cleaved by LC/A. It should be noted that we have not yet evaluated the functional rescue of synaptic signaling or the regeneration of normal synaptic morphologies with appropriate localization of synaptic proteins. The significant decrease in total protein recovered argues that some degree of neurotoxicity has occurred, and this is corroborated by morphological changes. It may be that most of the existing synapses have been disrupted in such a fashion to inactivate LC/A, or that the LC/A-intoxicated synapses are more sensitive to LTX-treatment. The data does suggest that LTX treatment results in calcium ‘overload’ and the abnormal flux of other ions through non-selective, cationic pores in the synaptic membrane, any of which may prove injurious to neurons [31,36]. The profound and sustained increase in cytosolic Ca^2+^ observed in ESNs followed by acute evidence of axodendritic varicosities is characteristic of excitotoxicity and presents a possible mechanism for clearance or inactivation of LC/A from synaptic termini via synaptic degeneration. Whether such degeneration results in the physical loss of LC/A from the synaptic compartment or in accelerated degradation of synapse-localized proteins via cellular proteosomal clearance mechanisms is unknown, but clearly of interest. Another possibility is that the significant increase in intracellular Ca^2+^ more directly inhibits the BoNT/A light chain cannot be discounted based on these data; e.g., through induction of autocatalysis [37]. 

#### 3.2.3. Is Neuronal Degeneration and/or Excitotoxicity Responsible for the Loss of BoNT Persistence?

Recovery from botulinum requires the re-innervation of the inactive muscle fiber, whether by regeneration of the original endplate or by establishment of a new endplate via axonal sprouting. *In vivo*, it appears that the endplate disassembles and the presynaptic compartment regresses in response to BoNT intoxication [38]. Shortly afterward, new axonal sprouts develop and attempt to re-innervate the target muscle fiber. The original endplate is eventually regenerated once the light chain has been cleared from the presynaptic terminal and appears to regain control as the primary efferent to the muscle fiber. The coordination of these events demonstrates the importance of synaptic remodeling in recovery of normal synaptic function after intoxication. It may be possible that by disrupting the integrity of the original synaptic terminal, BoNT light chains no longer remain associated with intoxicated synapses, thereby enabling the immediate re-innervation of endplates by re-activated nerve terminals. Similarly, *in vivo* studies have demonstrated dramatically accelerated recovery from paralysis following BoNT intoxication following nerve crush injury [26,27]. If this general approach proves to be efficacious in facilitating recovery from intoxication *in vivo*, then induced regeneration of paralyzed synapses could offer a novel, serotype-independent therapy for intoxication by persistent BoNTs.

The formation of varicosities in response to LTX treatment is suggestive of the morphological changes described following application of excitotoxic stimuli to cultured neurons. For example, glutamate treatment leads to varicosity formation in glutamatergic dendrites, whereas veratridine inactivation of voltage-gated sodium channels causes varicosity formation in both axons and dendrites. Under conditions of a sublethal excitotoxic stimulus, these varicosities have proven to be reversible via volume recovery pathways, although it is not known if axons and dendrites utilize similar mechanisms to resolve neuronal swelling [39,40,41]. While the nature of the potential relationship between excitotoxicity resolution and loss of BoNT persistence is still unclear, these results indicate that ESNs provide a stable cell-based platform to further interrogate the effect of LTX-induced synaptic regeneration on BoNT-persistence in cultured neurons. 

#### 3.2.4. ESNs Provide a Responsive, Genetically Tractable Model for LTX Research

There are several aspects of LTX activity that are not well understood [10]. For example, in addition to the formation of cation-selective pores permeable for Ca^2+^, LTX mutants incapable of forming pores can still induce membrane depolarization through inhibition of voltage-gated potassium channels and activation of L-type Ca^2+^ channels [42,43]. Whereas synaptic exocytosis induced by this mode of LTX action is dependent on the presence of the classical SNARE machinery, there is evidence that substantial levels of neurotransmitter release induced by pore-mediated Ca^2+^ internalization can still occur despite the absence of SNAP-25, VAMP-2 or Munc-13 [16]. Whether this involves a novel complex that can effect exocytosis at high Ca^2+^ concentrations or has a more mundane explanation such as enhanced reversal of membrane-associated amino acid transporters in response to changes in cytoplasmic ion concentrations or ATP-depletion is not known [44,45]. However, here we have demonstrated that ESNs offer an LTX-sensitive, neuron-based platform that has the potential to replace the use of primary neurons and neurogenic cells in studying the molecular, cellular and functional consequences of intoxication by LTX. As we have previously described for BoNTs, the additional capabilities of genetic tractability, neuronal subtype homogeneity and lot-to-lot consistency mean that the ESN model may also be a transformative tool for LTX research [46].

## 4. Conclusions

The finding that LTX treatment of BoNT/A-intoxicated ESNs rescued full-length SNAP-25 expression is the first demonstration of a successful therapeutic application in derived neurons. Although LTX treatment may not be a viable clinical therapy due to its broad activity, these data demonstrate that the general approach of induced synaptic degeneration may accelerate neuronal recovery from BoNT intoxication. Furthermore, they also suggest that LTX may be the active moiety in demonstrations that injection of paralyzed EDL muscles with crude homogenates from black widow spider venom glands facilitates recovery of muscle tension [25]. Finally, this work demonstrates that ESNs are a valid research tool for study of the mode of action of multiple neurotoxins, and thus may provide a transformation research platform for neurotoxin research and drug discovery.
